# Correlation of RAS-Pathway Mutations and Spontaneous Myeloid Colony Growth with Progression and Transformation in Chronic Myelomonocytic Leukemia—A Retrospective Analysis in 337 Patients

**DOI:** 10.3390/ijms21083025

**Published:** 2020-04-24

**Authors:** Klaus Geissler, Eva Jäger, Agnes Barna, Michael Gurbisz, Temeida Graf, Elmir Graf, Thomas Nösslinger, Michael Pfeilstöcker, Heinz Tüchler, Thamer Sliwa, Felix Keil, Christoph Geissler, Sonja Heibl, Josef Thaler, Sigrid Machherndl-Spandl, Otto Zach, Ansgar Weltermann, Peter Bettelheim, Reinhard Stauder, Armin Zebisch, Heinz Sill, Ilse Schwarzinger, Bruno Schneeweiss, Leopold Öhler, Ernst Ulsperger, Rajko Kusec, Ulrich Germing, Wolfgang R. Sperr, Paul Knöbl, Ulrich Jäger, Gregor Hörmann, Peter Valent

**Affiliations:** 1 Medical School, Sigmund Freud University, 1020 Vienna, Austria; 2 Department of Internal Medicine V with Hematology, Oncology and Palliative Medicine, Hospital Hietzing, 1130 Vienna, Austria; forschung.hietzing@gmail.com (T.G.); hietzing.forschung@gmail.com (E.G.); 3 Department of Laboratory Medicine, Medical University of Vienna, 1090 Vienna, Austria; eva.jaeger@akhwien.at (E.J.); michael.gurbisz@meduniwien.ac.at (M.G.); ilse.schwarzinger@meduniwien.ac.at (I.S.); 4 Blood Transfusion Service, Blood Transfusion Service for Upper Austria, Austrian Red Cross, 4020 Linz, Austria; agnes.barna@o.roteskreuz.at; 5 Department of Internal Medicine III, Hanusch Hospital, 1140 Vienna, Austria; thomas.noesslinger@wgkk.at (T.N.); michael.pfeilstoecker@wgkk.at (M.P.); tuechler@stht.at (H.T.); thamersliwa@gmail.com (T.S.); felix.keil@wgkk.at (F.K.); 6 Department of Laboratory Medicine, Hospital Hietzing, 1130 Vienna, Austria; christoph.geissler@wienkav.at; 7 Department of Internal Medicine IV, Hospital Wels-Grieskirchen, 4600 Wels, Austria; sonja.heibl@klinikum-wegr.at (S.H.); josef.thaler@klinikum-wels.at (J.T.); 8 Department of Internal Medicine I with Hematology with Stem Cell Transplantation, Hemostaseology and Medical Oncology, Ordensklinikum Linz Barmherzige Schwestern - Elisabethinen, 4020 Linz, Austria; Sigrid.Machherndl-Spandl@elisabethinen.or.at (S.M.-S.); Otto.Zach@Ordensklinikum.at (O.Z.); ansgar.weltermann@elisabethinen.or.at (A.W.); peter@bettelheim.eu (P.B.); 9 Internal Medicine V with Hematology and Oncology, Medical University of Innsbruck, 6020 Innsbruck, Austria; reinhard.stauder@i-med.ac.at; 10 Department of Internal Medicine, Division of Hematology, Medical University of Graz, 8036 Graz, Austria; armin.zebisch@medunigraz.at (A.Z.); heinz.sill@medunigraz.at (H.S.); 11 Otto-Loewi-Research Center for Vascular Biology, Immunology and Inflammation, Division of Pharmacology, Medical University of Graz, 8036 Graz, Austria; 12 Department of Internal Medicine, Hospital Kirchdorf, 4560 Kirchdorf, Austria; Bruno.Schneeweiss@gespag.at; 13 Department of Internal Medicine/Oncology, St. Josef Hospital, 1130 Vienna, Austria; leopold.oehler@sjk-wien.at; 14 Department of Internal Medicine, Hospital Horn, 3580 Horn, Austria; ernst.ulsperger@aon.at; 15 School of Medicine, University of Zagreb, University Hospital Dubrava, 10000 Zagreb, Croatia; Rajko.Kusec@irb.hr; 16 Department of Hematology, Oncology, and Clinical Immunology, Heinrich-Heine-University, 40225 Düsseldorf, Germany; germing@med.uni-duesseldorf.de; 17 Department of Internal Medicine I, Division of Hematology and Hemostaseology, Medical University of Vienna, 1090 Vienna, Austria; wolfgang.r.sperr@meduniwien.ac.at (W.R.S.); paul.knoebl@meduniwien.ac.at (P.K.); ulrich.jaeger@meduniwien.ac.at (U.J.); peter.valent@meduniwien.ac.at (P.V.); 18 Central Institute of Medical and Chemical Laboratory Diagnostics, Medical University of Innsbruck, 6020 Innsbruck, Austria; gregor.hoermann@meduniwien.ac.at; 19 Ludwig Boltzmann Institute for Hematology and Oncology (LBI HO), Medical University of Vienna, 1090 Vienna, Austria

**Keywords:** CMML, AML, RAS-pathway mutations, CFU-GM

## Abstract

Although the RAS-pathway has been implicated as an important driver in the pathogenesis of chronic myelomonocytic leukemia (CMML) a comprehensive study including molecular and functional analyses in patients with progression and transformation has not been performed. A close correlation between RASopathy gene mutations and spontaneous in vitro myeloid colony (CFU-GM) growth in CMML has been described. Molecular and/or functional analyses were performed in three cohorts of 337 CMML patients: in patients without (A, *n* = 236) and with (B, *n* = 61) progression/transformation during follow-up, and in patients already transformed at the time of sampling (C, *n* = 40 + 26 who were before in B). The frequencies of RAS-pathway mutations (variant allele frequency ≥ 20%) in cohorts A, B, and C were 30%, 47%, and 71% (*p* < 0.0001), and of high colony growth (≥20/10^5^ peripheral blood mononuclear cells) 31%, 44%, and 80% (*p* < 0.0001), respectively. Increases in allele burden of RAS-pathway mutations and in numbers of spontaneously formed CFU-GM before and after transformation could be shown in individual patients. Finally, the presence of mutations in RASopathy genes as well as the presence of high colony growth prior to transformation was significantly associated with an increased risk of acute myeloid leukemia (AML) development. Together, RAS-pathway mutations in CMML correlate with an augmented autonomous expansion of neoplastic precursor cells and indicate an increased risk of AML development which may be relevant for targeted treatment strategies.

## 1. Introduction

Chronic myelomonocytic leukemia (CMML) is a hematopoietic malignancy of the elderly that is characterized by overlapping features of myelodysplastic syndromes (MDS) and myeloproliferative neoplasms (MPN) and an inherent risk of transformation to secondary acute myeloid leukemia (sAML) [1,2,3,4,5,6,7]. The median overall survival of CMML patients is approximately 30 months. Allogeneic hematopoietic stem cell transplantation is the only curative therapy approach, but this form of treatment is only rarely feasible because of age and/or comorbidities that are often found in patients with CMML. In patients ineligible for transplantation, intensive chemotherapy results in low response rates and short response duration. Hydroxyurea is commonly used as palliative drug to control myeloproliferation in advanced CMML and sAML [8]. The cytidine analogues azacitidine (AZA) and decitabine have demonstrated some efficacy in delaying disease progression in advanced CMML and have been approved for the treatment of this disease [9,10]. Since many patients eventually die from transformation and/or progression of disease a better understanding of disease evolution is crucial to improve treatment and prognosis.

At the molecular level CMML is a heterogeneous disease similar to other hematologic neoplasms. However, many of the gene alterations detected in CMML affect similar or the same oncogenic machineries and signaling pathways [11]. Over the past few years, a large number of mutations in genes encoding epigenetic regulators (*TET2*, *ASXL1*, *EZH2*, *UTX*, *IDH1*, *IDH2*, and *DNMT3A*), splicing factors (*SF3B1*, *SRSF2*, *ZRSF2*, and *U2AF1*), and signaling molecules (*NRAS*, *KRAS*, *CBL*, *JAK2*, and *FLT3*) have been identified in clonal cells in CMML [12]. In a few patients, progression of CMML was associated with an increment of *RAS* mutational burden, suggesting a role of RAS-pathway hyperactivation in progression and transformation to AML [13]. Although the impact of RAS-pathway mutations in the risk to develop secondary leukemia has been studied in larger cohorts of patients with CMML, patients who had already transformed to AML were usually not included and thus these studies could not provide an answer to the question to what extent the RAS-pathway can indeed contribute to transformation [14,15,16]. In one study, the molecular features and mutational patterns were analyzed during blast transformation of CMML and the RAS-pathway was apparently involved [17]. In none of these studies, however, functional tests of RAS-pathway hyperactivation were applied.

In juvenile myelomonocytic leukemia (JMML), a RAS-pathway driven hematologic malignancy in children, growth factor-independent formation of granulocyte/macrophage colony-forming units (CFU-GM) in semisolid cultures is considered a hallmark of the disease. Therefore, autonomous CFU-GM formation has been included as a diagnostic criterion in previous WHO classifications [2,4]. If considering this test as a functional parameter of RAS-pathway hyperactivation indications for aberrant RAS-pathway signaling in CMML can actually be traced back for 30 years when we described this in vitro phenomenon in 1988 in a subset of our CMML patients [18]. Later, we have shown that spontaneous CFU-GM formation in CMML is a GM-CSF-related in vitro phenomenon [19]. It has also been described that CMML progenitors are hypersensitive against GM-CSF in a study of Padron et al. [20]. In preclinical mouse models, molecular alterations of RASopathy genes in murine hematopoietic cells are leading to a myelomonocytic leukemia like phenotype in vivo and to spontaneous myeloid colony formation due to GM-CSF hypersensitivity in vitro [21,22,23,24,25]. Recently we were able to demonstrate a close correlation between increased spontaneous colony formation in CMML patients and the presence of RAS-pathway mutations [26]. Together these findings strongly suggest that high spontaneous in vitro CFU-GM formation in CMML reflect RAS-pathway hyperactivation at a functional level. Although a correlation of mutations in RAS-pathway genes and spontaneous myeloid colony formation has been shown by us in CMML patients without transformation, a comprehensive analysis of the RAS-pathway in patients with CMML derived AML has not been performed. Molecular as well as functional data on the RAS-pathway aberrations, however, in this particular cohort would be of significant interest considering the dismal prognosis of patients and the availability of RAS pathway inhibitors.

In the “Austrian Biodatabase for Chronic Myelomonocytic Leukemia” (ABCMML) we retrospectively and prospectively collect hematological, clinical, molecular, and biological information of patients with CMML from different centers in a real world setting [27]. Due to the retrospective character of our database it contains data that are obtained from patients being in different phases of CMML evolution at the time of inclusion and subsequent follow up. Therefore, we divided patients into three cohorts based on criteria recently proposed by an international consortium: [28] patients without evidence of progression (cohort A), patients who developed disease progression (transformation and/or disease-related death) during follow up (cohort B), and patients who had already transformed to sAML at the time of sampling (cohort C). Using data (molecular, *n* = 313; CFU-GM, *n* = 196) from 337 CMML patients we compared the frequencies of RASopathy gene mutations (variant allele frequency (VAF) ≥ 20%) and of high CFU-GM growth (≥20/10^5^ peripheral blood mononuclear cells (PBMNC)) in patient cohorts A, B and C and were able to monitor disease evolution in individual patients in whom serial samples were available.

## 2. Results

### 2.1. Impact of Disease Stage on Survival in Patients with CMML

The total cohort comprised 209 (62%) men and 128 (38%) women with a median age of 73.0 years (range: 36–93 years) which is comparable to other cohorts reported in the literature [14,15,16,17]. As mentioned above the patients were grouped into one of three categories: patients without evidence of AML development and/or progression related death at any time (cohort A, *n* = 236), patients who developed AML and/or progression related death during follow up (cohort B, *n* = 61) and patients after transformation to sAML (cohort C, *n* = 40). In order to demonstrate the clinical significance of this categorization we first calculated Kaplan Meier plots for each of the patient categories. As shown in supplemental Figure S1 there was a clear discrimination between the three categories with a median survival of 30 months in cohort A, 21 months in cohort B, and 5 months in cohort C (*p* < 0.0001). These finding suggest that the three categories that we have chosen in this study really represent different stages of CMML evolution.

### 2.2. Hematologic and Cytogenetic Characteristics in Patient-Subgroups

The laboratory characteristics of these patient groups are shown in Supplementary Table S1, including white blood count (WBC), hemoglobin (Hb) level, platelet count, percentage of PB blasts, and percentage of monocytes. As compared to CMML patients without evidence of transformation and/or disease-related death at any time (cohort A) patients who had already transformed to sAML (cohort C) had significantly (<0.05) higher WBC counts and PB blast cell percentages, and significantly lower Hb levels and platelet counts. The WBC counts and PB blast cell percentages were also significantly higher in CMML patients who later progressed during follow up (group B) than in patients of group A. However, cohort B was not significantly different to cohort A with regard to Hb levels, and platelet counts. Since group B was relatively small we cannot exclude the possibility that significant differences would have been found in this category when the number of these patients would have been larger. The frequencies of high risk cytogenetic abnormalities such as + 8, −7/del(7q), and complex karyotype (≥3 abnormalities) in cytogenetic analyses were 16% (19/122) in cohort A, 41% (16/39) in cohort B, and 30% (8/27) in cohort C (*p* = 0.003).

### 2.3. RAS-Pathway Mutations Correlate with AML Evolution in Patients with CMML

**Figure 1:**
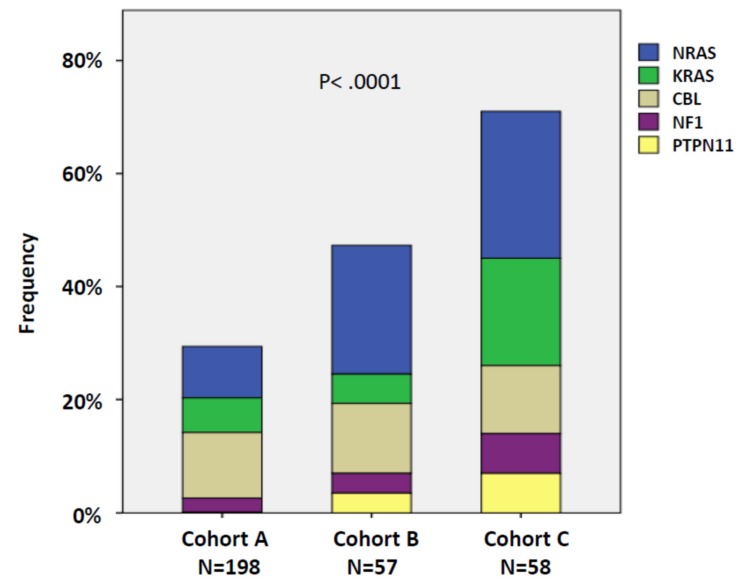
Frequencies of RASopathy gene mutations in the 3 patient cohorts: patients without evidence of progression (cohort A), patients who developed disease progression (transformation and/or disease-related death) during follow up (cohort B), and patients who had already transformed to secondary acute myeloid leukemia (AML) at the time of sampling (cohort C). Individual genes are indicated by different colors. Cohort C includes 18 patients from cohort B who initially had no evidence of transformation but developed AML during observation. In patients with more than one mutation in RASopathy genes the mutation with the highest variant allele frequency (VAF) was used for this analysis.

Molecular analysis by NGS was performed in 295 patients. Because of their functional significance RAS signaling pathway components including *NRAS, KRAS, NF-1, PTPN11,* and *CBL* were summarized as RASopathy genes within one category. As shown in Figure 1 there was a continuous increase in the frequencies of RAS-pathway mutations in patients who never developed AML (A) to patients with overt transformation, with 30% (59/198) in cohort A, 47% (27/57) in cohort B, and 71% (41/58) in cohort C, respectively (*p* < 0.0001; A vs. B, *p* = 0.134; B vs. C, *p* = 0.011). The proportions of mutations of individual genes can be seen in the stacked histogram. The numbers for the different cohorts and genes, respectively, were: cohort A: NRAS 9.1%, KRAS 6.1%, CBL 11.6%, NF1 2.5%, and PTPN11 0.5%; cohort B: NRAS 22.8%, KRAS 5.3%, CBL 12.3%, NF1 3.5%, and PTPN11 3.5%; cohort C: NRAS 25.9%, KRAS 19.0%, CBL 12.0%, NF1 7.0%, and PTPN11 7.0%.

Supplementary Table S2 shows the frequencies of non-RASopathy gene mutations with a frequency of at least 10% in patient samples. There was a significant increase in the frequency of *RUNX1* mutations towards progression/transformation of CMML into AML but no increase in the prevalence of mutations of *SETBP1, TET2, EZH2, ASXL1, SRSF2,* and *TP53.*

**Figure 2:**
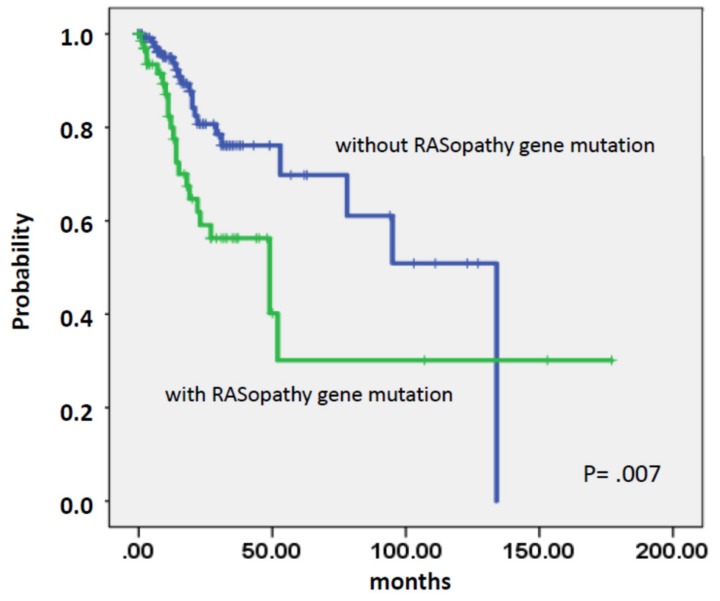
Time to AML transformation in chronic myelomonocytic leukemia (CMML) patients stratified by the presence or absence of RASopathy gene mutations.

As shown in Figure 2 the presence of mutations in RASopathy genes prior to transformation was associated with an increased risk of AML development (*p* = 0.007).

**Figure 3:**
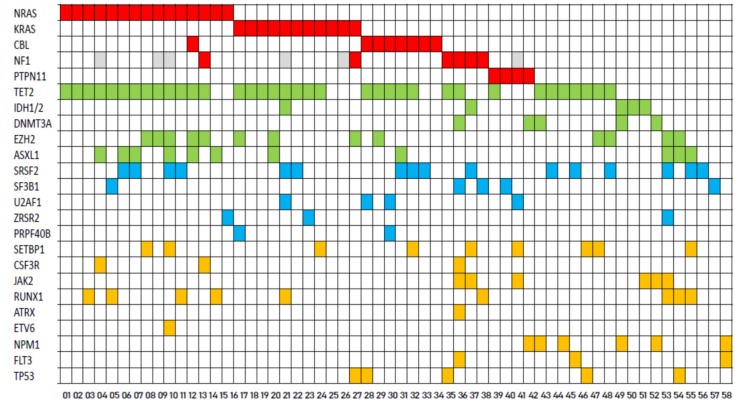
Comprehensive mutation status of genes in patients with CMML derived AML. Each column corresponds to one patient. Colored squares indicate mutated, white squares wild-type genes. The colors of mutant genes indicate the most affected functional categories. Red, green, blue, and yellow represent the RAS-pathway, epigenetic regulators, spliceosome, and other components, respectively. Missing data are indicated by gray squares.

**Figure 4:**
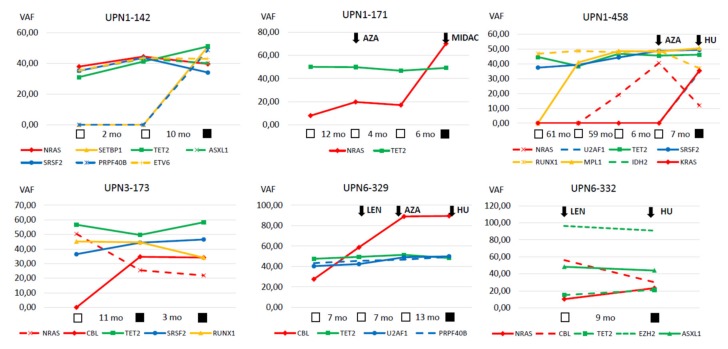
Serial mutation analysis in individual patients. The variant allele frequencies of 6 individual patients are shown at different time points during the course of their disease. Open squares indicate time points pre transformation and closed squares time points after transformation. Initiation of treatment is indicated by arrows: AZA—azacitidine, HU—hydoxyurea, LEN—lenalidomide, and MIDAC—mitoxantrone + cytarabine.

A comprehensive mutation status of CMML patients with sAML (cohort C) is shown in Figure 3. Among RAS-pathway-related mutations *NRAS* mutations (*n* = 15) were the most common ones followed by *KRAS* (*n* = 11), *CBL* (*n* = 8), *NF1* (*n* = 6), and *PTPN11* (*n* = 4), respectively. Detailed information regarding the variants detected in these samples are given in Supplementary Table S3. In six patients, serial samples during progression were available to perform NGS analysis (Figure 4). In five/six patients, there was an increase by more than two-fold in the allele burden of RAS-pathway aberrations during disease progression/transformation.

### 2.4. High Autonomous in Vitro Colony Formation Correlates with AML Evolution in Patients with CMML

**Figure 5:**
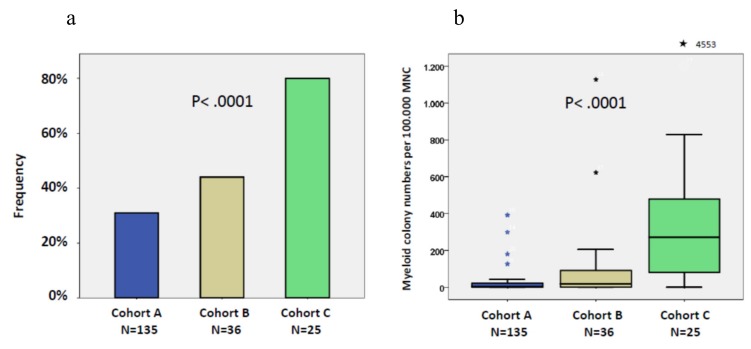
(**a**) Frequencies of high spontaneous myeloid colony formation (>20/10^5^ peripheral blood mononuclear cells (PBMNC)) in patients without evidence of progression (cohort A), patients who developed disease progression (transformation and/or disease-related death) during follow up (cohort B), and patients who had already transformed to secondary AML at the time of sampling (cohort C). Cohort C includes 13 patients from cohort B who initially had no evidence of transformation but developed AML during observation. (**b**) Box plots showing the distribution of spontaneous colony numbers in the 3 patient cohorts including median values, minimum values, maximum values, as well as upper and lower quartiles, respectively. Cultures were plated in duplicates or triplicates, respectively, at 25–100 × 10^3^ PBMNC/mL. Aggregates with more than 40 translucent, dispersed cells were counted as CFU-GM. CFU-GM data from patients are expressed as mean values from cultures.

In vitro cultures were performed in 183 patients with CMML. Recently we demonstrated a close correlation between high spontaneous colony formation in CMML patients and the presence of RAS-pathway mutations [26]. This correlation was also seen in this study using 162 samples in which both NGS data and colony data were available. The median number of spontaneously formed CFU-GM/10^5^ MNC was 47 (range 0–1127) in RAS-positive patients as compared to 4 (0–812) in RAS-negative patients (*p* < 0.0001). Unstimulated in vitro myeloid colony formation in RAS-positive CMML patients is also much higher than the spontaneous formation of CFU-GM in normal individuals (median 4.8/10^5^ PBMNC, range 3.5–8.5), which has been reported by us previously [29]. The frequency of spontaneous CFU-GM growth ≥0/10^5^ in the 3 patient cohorts is shown in Figure 5a. There was a continuous increase in the frequency of high spontaneous myeloid colony formation (≥20/10^5^ PBMNCs) from patients in group A (42/135, 31%) to patients in group B (16/36, 44%) and to patients in group C (20/25, 80%), respectively (*p* < 0.0001; A vs. B, *p* = 0.141; B vs. C, *p* = 0.005). The boxplot in Figure 5b shows a large variation in colony numbers between single patients in the different cohorts, however, median CFU-GM numbers per 10^5^ MNC clearly increased with 4.5 in cohort A, 19 in cohort B, and 287 in cohort C (*p* < 0.0001).

**Figure 6:**
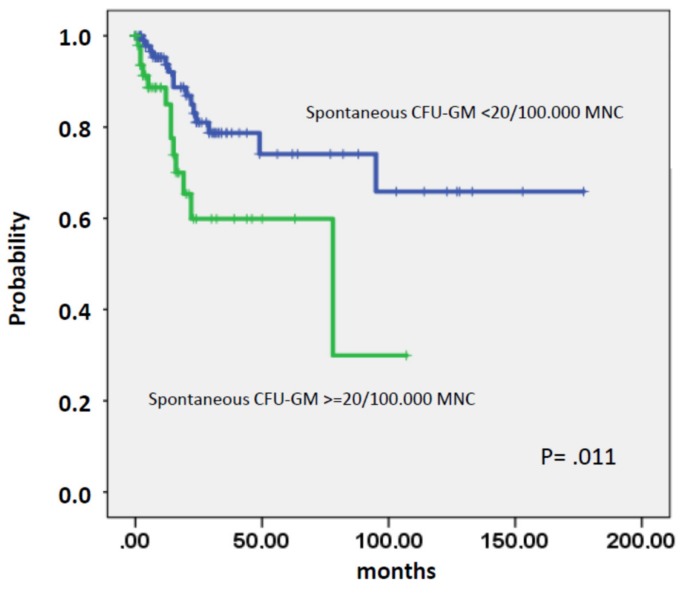
Time to AML transformation in CMML patients stratified by the presence or absence of spontaneous CFU-GM growth >20/10^5^ mononuclear cells.

As shown in Figure 6 the presence of high spontaneous CFU-GM in patients prior to transformation was associated with an increased risk of AML development (*p* = 0.011).

Table 1 shows the numbers of spontaneously formed CFU-GM in eight patients in whom in vitro cultures could be performed before and after transformation to sAML. Seven of eight patients were characterized by NGS, and in all of them mutations of RASopathy genes were detected (*NRAS* 4, *KRAS* 1, *CBL* 1, *PTPN11* 1). As compared to pre transformation values, a marked increase of colony numbers following transformation to sAML can be seen in all eight patients (median [range] CFU-GM/10^5^ MNC pre transformation 41.5 (1–622), post transformation 263 (48–4553); *p* = 0.012).

## 3. Discussion

CMML is an incurable stem cell-derived MDS/MPN overlap neoplasm characterized by monocyte expansion and an increased risk to transform to AML [1,2,3,4,5,6,7]. Although the RAS pathway has been implicated in the pathogenesis of CMML, little is known about mechanistic and functional correlates and how progression impacts survival in these patients. We have analyzed data from CMML patients collected in our ABCMML-registry and found that RAS-pathway mutations and thus RAS hyperactivation correlates with autonomous expansion of neoplastic stem/progenitor cells and with disease progression. Moreover, we show that the allelic burden of RAS-pathway-related mutations increase during progression in individual patients with CMML. The frequency of RAS-pathway mutations in our patients were within the range of other published series in which marked variations can be found ranging from 23%–60% [30,31,32]. Reasons for these variations may be differences in the criteria applied for CMML diagnosis, differences in disease stages investigated, and differences in the number of target genes/regions covered by the NGS panels applied. In this regard, it is worth noting that the diagnostic criteria for CMML have significantly changed during the last three decades, in particular when comparing the former FAB classification with the most recent WHO classification of CMML [1,2,3,4,5]. In view of the lack of specific disease markers, the diagnosis of CMML is still based primarily on clinical parameters and morphologic features of clonal cells. The hybrid nature of CMML with features of both a MDS and a MPN creates also diagnostic difficulties. A most important diagnostic parameter is the presence of PB monocytosis which is a prerequisite diagnostic criterion of CMML [7,33]. Previous observations have already implicated the RAS-pathway in the pathogenesis of CMML [12]. Our data confirm this notion and provide evidence that RAS pathway mutations are critical involved in CMML evolution and progression.

The frequency of RASopathy genes in our patients was clearly increasing during progression and was observed in more the 70% of all patients with CMML-derived sAML. In six of these patients, serial mutation analyses could be performed. The fact that VAFs of RASopathy genes in all of these cases were lower at first presentation than VAFs at the time of AML and in general lower than VAFs of epigenetic and splicing factors suggests that mutations in genes relevant to the RAS signaling pathway were later acquired and were driving events for disease progression. These data are in agreement with data from others demonstrating that mutations of critical genes involved in signaling cascades, including the RAS-pathway, were also acquired as late events in MDS [34,35,36,37]. Moreover, recent preclinical models suggest that activating *RAS* mutations and somatic loss-of-function mutations in TET2 exert cooperating effects and accelerated disease progression [38,39].

During the progression of CMML to AML spontaneous CFU-GM formation was found to be continuously increasing. There is now sufficient evidence to consider spontaneous CFU-GM formation as a functional test of RAS-pathway hyperactivation in CMML [26]. Due to the fact that in CMML more than one functionally relevant molecular aberration can be detected in many patients we think that the spontaneous in vitro colony formation of CFU-GM may help predict that the RAS-pathway is involved in disease evolution which may in turn have clinical (prognostic and therapeutic) implications. Another functional assay for RAS-pathway hyperactivity may be an increased expression of phosphorylated STAT5 which is a downstream target of the RAS-pathway. Although we did not perform such experiments in this study, Padron et al. have demonstrated that neoplastic cells in CMML exhibit increased STAT5 activity [20].

We confirm previous studies which have reported that RAS-pathway mutations are common in CMML but are usually rare in other myeloid malignancies such as CML or Ph-negative MPN [30]. In one study, *NRAS* mutations were not found in any of 86 CML patients in blast crisis examined [40]. Only one patient, in whom the initial diagnosis of CML blast crisis had been revised to CMML, displayed an *NRAS* mutation within codon 13 [40]. In another study in which targeted cancer exome sequencing was performed in BCR-ABL-negative MPNs, *NRAS* mutations were found in only 4.7% of 168 patients with primary myelofibrosis (MF) and in none of the patients with polycythemia vera (PV) [41].

In addition to mutations of the RAS-pathway, a large number of mutations in other genes were found in our study which is in line with previous reports [12]. In contrast to RAS-pathway mutations, however, aberrations in the majority of other genes were not significantly different in our three patient groups. This argues against a major role of these aberrations in the transformation process although contributions of such mutations in individual patients cannot be excluded. Significant changes were only observed for *RUNX1*. A trend towards a higher risk to transform to AML was reported for CMML patients with *RUNX1* mutated cells, especially patients with C-terminal mutations [42]. An association between *ASXL1* mutations and acute transformation in CMML has been described but there was only a trend found in our study [43].

We are aware of the limitations of our study. For example, most of the information used in this study was derived from real world data that were not collected systematically or prospectively. In addition, data from patient records were obtained over many years and from many different centers. However, real world data have recently been recognized as an important way to get insights into the natural history of rare diseases [44]. CMML is a rare disease and adequate patient numbers for a systematic and prospective study are not easy to collect within a limited time frame. In a substantial number of patients we had serial samples from several time points giving us the possibility to determine the variant allele frequencies of gene mutations at different time points during the course of their disease. Since many patients had already died when we started our analyses, germline controls were not available in most patients. However, almost all mutations in RASopathy genes which were found in our study have already been reported as somatic aberrations, and the allelic burden of the mutant forms detected in our patients also argue against germline defects.

Our clinical data confirm the dismal prognosis of CMML patients who transform to AML. Such patients had a median survival of only 5.0 months which is in accordance with other published series [45]. These data clearly indicate the medical need for improving treatment concepts for these patients. The findings of this study may contribute to achieve this goal by suggesting that hyperactivation of the RAS signaling pathway plays a major role in the transformation process from CMML into AML. The study of RAS-pathway inhibitors seems to be a logical step in our efforts. In fact, a recent study has investigated the effect of such an inhibitor in patients with RAS-pathway driven hematological malignancies including CMML and has shown some responses in these poor risk patients who are extremely difficult to treat especially when they progress to AML [46].

## 4. Patients and Methods

### 4.1. Patients

We employed data from 337 CMML patients from the ABCMML which has been shown to be a representative and useful real-life data source for biomedical research [27]. In this database, we retrospectively collected epidemiologic, hematologic, biochemical, clinical, immunophenotypic, cytogenetic, molecular and biologic data of patients with CMML from different centers. Internal Review Board approval was obtained at each institution (ethics committee of the city of Vienna, EK 15-059-VK). Clinical and laboratory routine parameters were obtained from patient records. A detailed central manual retrospective chart review was carried out to ensure data quality before analysis of data from institutions. Data curation included the extraction of discrete data elements from patient records, a check for accuracy and consistency of data, and a verification that baseline data were reflective of CMML that was strictly defined according to WHO criteria. Progression and/or transformation was defined as disease related mortality, transformation to AML, or a combination of criteria as noted by Savona et al. [28]. Briefly the proposed criteria for disease progression include major criteria such as increase in blast count, evidence of cytogenetic evolution, new extramedullary disease and minor criteria such as transfusion dependence, significant loss of maximal response on cytopenias, reduction in Hb by ≥1.5 g/dL from best response or from baseline, increasing symptoms, and evidence of clonal evolution, respectively. A combination of 2 major criteria, 1 major and 2 minor criteria, or 3 minor criteria have to be met. By using these criteria our study population was categorized in patients without (A, *n* = 236) and with (B, *n* = 61) progression/transformation during follow-up, and in patients already transformed at the time of sampling (C, *n* = 40 + 26 who were before in B). Clinical and laboratory routine parameters were obtained from the patients’ records. Blood counts were taken at the time of sampling and survival analysis was calculated from the sampling date.

The specimens investigated in order to analyze the frequencies of RASopathy gene mutations included individual samples obtained from 277 patients, and two serial samples, one at the time before (group B) and another after transformation (group C) from 18 patients, respectively. Likewise, the specimens investigated in order to analyze the frequencies of high colony growth included individual samples obtained from 170 patients, and two serial samples, one at the time before (group B) and another after transformation (group C) from 13 patients, respectively.

### 4.2. Cytogenetic Analysis

Cytogenetic studies were performed using G-banding according to standard techniques on BM cells for 24–48 h in unstimulated culture. Chromosome aberrations were classified according to the International System for Human Cytogenetic Nomenclature (ISCN). CMML-specific cytogenetic risk classification was low for normal karyotype and isolated -Y, intermediate for other abnormalities and high for trisomy 8, complex karyotype (≥3 abnormalities), and abnormalities of chromosome 7 [47]. In general samples were taken before any disease-modifying treatment such as allogeneic stem cell transplantation, aggressive chemotherapy, or hypomethylating agents.

### 4.3. Molecular Studies

Genomic DNA was isolated from mononuclear cell (MNC) fractions of these blood samples according to standard procedures. The mutational status of CMML-related protein coding genes was determined by targeted amplicon sequencing using the MiSeq platform (Illumina, San Diego, CA, USA). Details regarding gene panel, library preparation and data processing have been reported previously [27]. To minimize the chance to capture mutations which may be associated with non-neoplastic conditions we chose a VAF cutoff of ≥20% which has been shown to provide a specificity of 86% [48].

### 4.4. Colony Assay

In one of our centers (Medical University of Vienna) the assessment of hematopoietic colony formation in vitro has been an integral part of the diagnostic work up in patients with suspected myeloid malignancies for many years [49]. CFU-GM growth was assessed in semisolid cultures without growth factors as previously described [19]. A detailed description of the technique is provided in the Supplementary Materials.

### 4.5. Statistical Analysis

The log-rank test was used to determine whether individual parameters were associated with overall survival (OS) and time to AML transformation. OS was defined as the time from sampling to death (uncensored) or last follow up (censored). Time to AML transformation was defined as the time of sampling to the time of transformation to secondary AML (uncensored) or death/last contact (censored). Dichotomous variables were compared between different groups with the use of the chi-square test. The Mann-Whitney-U-test was used to compare 2 and the Kruskal–Wallis test to compare more than 2 unmatched groups when continuous variables were not normally distributed. Results were considered significant at *p* < 0.05. Statistical analyses were performed with the SPSS version 19.0.0 (SPSS Inc, Chicago, Illinois, USA); the reported *p*-values were 2-sided.

## Figures and Tables

**Table 1:** White blood cell counts and numbers of spontaneously formed CFU-GM in 8 patients in whom in vitro cultures could be performed before and after transformation to secondary acute myeloid leukemia.

Patient	Genotype	WBC G/L Pretransform. (%PB blasts)	WBC G/L Posttransform. (%PB blasts)	CFU–GM/10^5^ MNC Pretransform.	CFU-GM/10^5^ MNC Posttransform.
UPN1-026	CBL	12.3 (2%)	178 (60%)	35	302
PN1-033	NRAS	7.8 (4%)	160 (10%)	200	533
UPN1-038	NA	93.8 (1%)	50.7 (30%)	622	4553
UPN1-071	PTPN11	74.0 (8%)	161 (18%)	11	48
UPN1-128	NRAS	57.5 (3%)	155 (13%)	59	272
UPN1-142	NRAS	20.3 (0%)	80.7 (1%)	48	202
UPN1-171	NRAS	55.5 (0%)	50.0 (54%)	1	254
UPN1-468	NRAS	8.5 (0%)	24.5 (57%)	8	381

NA—not available.

## Supplementary Materials

The following are available online at https://www.mdpi.com/1422-0067/21/8/3025/s1, Figure S1: Kaplan-Meier survival curves of the 3 patient cohorts. Table S1: Laboratory values of the 3 patient cohorts: patients without evidence of progression (cohort A), patients who developed disease progression (transformation and/or disease-related death) during follow up (cohort B), and patients who had already transformed to secondary AML at the time of sampling (cohort C). Table S2: Frequencies of other than RASopathy gene mutations in the 3 patient cohorts: patients without evidence of progression (cohort A), patients who developed disease progression (transformation and/or disease-related death) during follow up (cohort B), and patients who had already transformed to secondary AML at the time of sampling (cohort C). Table S3: Detailed informations (region, ENST, ENSP, variant allele frequency) of molecular aberrations detected in RASopathy genes in samples of patients with CMML derived AML.
