# Supplementary material for: Correlation of RAS-Pathway Mutations and Spontaneous Myeloid Colony Growth with Progression and Transformation in Chronic Myelomonocytic Leukemia—A Retrospective Analysis in 337 Patients
Source: Int J Mol Sci. 2020 Apr 24;21(8):3025. doi: 10.3390/ijms21083025 (PMC7215883; doi:10.3390/ijms21083025)
Supplement: Supplementary file 1 [file ijms-21-03025-s001.pdf]

**Supplementary figure 1.** Kaplan-Meier survival curves of the 3 patient cohorts. Patients were divided into 3 cohorts based on criteria recently proposed by an international consortium:[28] patients without evidence of progression (cohort A, n=236), patients who developed disease progression (transformation and/or disease-related death) during follow up (cohort B, n=61), and patients who had already transformed to sAML at the time of sampling (cohort C, n=40). Median survival was 30 months in cohort A, 21 months in cohort B, and 5 months in cohort C, respectively.

**Suppl. Fig. 1**

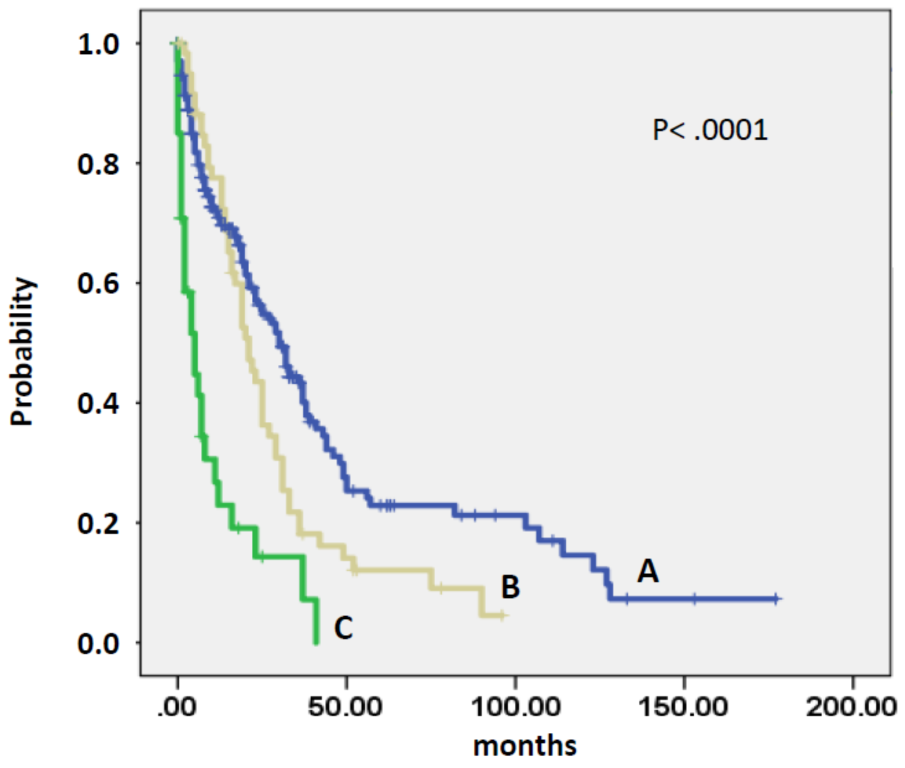

**Supplementary table 1.** Laboratory values of the 3 patient cohorts: patients without evidence of progression (cohort A), patients who developed disease progression (transformation and/or disease-related death) during follow up (cohort B), and patients who had already transformed to secondary AML at the time of sampling (cohort C).

| Parameters | Cohort A, N=236 | Cohort B, N=61 | Cohort C, N=40 |
|---|---|---|---|
| WBC x $10^9$/L; median (range) | 12.2 (2.5-139) | 17.9 (3.6-94) | 27.8 (4.1-205) |
| Hb g/dL; median (range) | 11.0 (4.3-14.9) | 11.1 (6.4-15.3) | 9.8 (4.1-14.1) |
| Platelet x $10^9$/L; median (range) | 115 (5-726) | 89 (7-695) | 56 (17-397) |
| PB Blast %; median (range) | 0 (0-17) | 0 (0-13) | 2.0 (0-94) |
| Monocyte %; median (range) | 22 (3-60) | 24 (4-74) | 26 (0-74) |

**Supplementary table 2.** Frequencies of other than RASopathy gene mutations in the 3 patient cohorts: patients without evidence of progression (cohort A), patients who developed disease progression (transformation and/or disease-related death) during follow up (cohort B), and patients who had already transformed to secondary AML at the time of sampling (cohort C). NGS analysis was performed as described in Patients and Methods. Details regarding gene panel, library preparation and data processing have been reported previously.[27] In case of conflicting results for the pathogenicity of a variant, the underlying data were manually rechecked. Variants were considered (likely) benign unless they satisfied all of the following conditions: the mutation occurred in a protein coding region, the mutation function was not synonymous, the annotation from ClinVar was not benign, and the change was not found at a frequency of 1% or higher in a population. Clearly pathogenic variants and variants of unknown significance were retained as potential mutations. Only mutations with allele frequencies of at least 20% were considered as positive, and only mutations with a frequency of at least 10% in the total cohort are shown.

| Genes | Cohort A | Cohort B | Cohort C | P |
|---|---|---|---|---|
| SETBP1 | 33/198 (17%) | 16/57 (28%) | 15/58 (26%) | .090 |
| TET2 | 145/198 (73%) | 35/57 (61%) | 39/58 (67%) | .202 |
| EZH2 | 35/198 (18%) | 9/57 (16%) | 12/58 (21%) | .784 |
| ASXL1 | 34/198 (17%) | 17/57 (30%) | 11/58 (19%) | .106 |
| SRSF2 | 76/198 (38%) | 19/57 (33%) | 17/58 (29%) | .409 |
| RUNX1 | 17/198 (9%) | 11/57 (19%) | 13/58 (22%) | .007 |
| TP53 | 21/198 (11%) | 8/57 (14%) | 5/58 (9%) | .635 |

**Supplementary table 3.** Detailed informations (region, ENST, ENSP, variant allele frequency) of molecular aberrations detected in RASopathy genes in samples of patients with CMML derived AML.

| Sample | Gene | Region | ENST | ENSP | VAF |
|---|---|---|---|---|---|
| 1 | *NRAS* | 115258748 | c.34G>A | p.Gly12Ser | 25.44 |
| 2 | *NRAS* | 115258748 | c.34G>C | p.Gly12Arg | 47.89 |
| 3 | *NRAS* | 115258747 | c.35G>A | p.Gly12Asp | 90.63 |
| 4 | *NRAS* | 115258748 | c.34G>A | p.Gly12Ser | 30.58 |
| 5 | *NRAS* | 115258748 | c.34G>C | p.Gly12Arg | 38.77 |
| 6 | *NRAS* | 115258748 | c.34G>C | p.Gly12Arg | 31.90 |
| 7 | *NRAS* | 115258744 | c.38G>A | p.Gly13Asp | 49.92 |
| 8 | *NRAS* | 115258747 | c.35G>A | p.Gly12Asp | 47.69 |
| 9 | *NRAS* | 115258748 | c.34G>T | p.Gly12Cys | 70.17 |
| 10 | *NRAS* | 115258744 | c.38G>T | p.Gly13Val | 44.51 |
| 11 | *NRAS* | 115256521 | c.190T>G | p.Tyr64Asp | 32.78 |
| 12 | *NRAS*<br>*CBL* | 115258747<br>119148922 | c.35G>A<br>c.1142G>C | p.Gly12Asp<br>p.Cys381Ser | 30.20<br>23.20 |
| 13 | *NRAS*<br>*NF1* | 115258744<br>29663388 | c.38G>A<br>c.4979T>A | p.Gly13Asp<br>p.Ile2015Asn | 36.22<br>35.22 |
| 14 | *NRAS* | 115258747 | c.35G>A | p.Gly12Asp | 45.60 |
| 15 | *NRAS* | 115258744 | c.38G>A | p.Gly13Asp | 28.40 |
| 16 | *KRAS* | 25398285 | c.34G>A | p.Gly12Ser | 21.72 |
| 17 | *KRAS* | 25398284 | c.35G>A | p.Gly12Asp | 41,46 |
| 18 | *KRAS* | 25398285 | c.34G>A | p.Gly12Ser | 47.74 |
| 19 | *KRAS* | 25380279 | c.179G> | p.Gly60Val | 49.14 |
| 20 | *KRAS* | 25398285 | c.34G>A | p.Gly12Ser | 34.24 |
| 21 | *KRAS* | 25380283 | c.183A>T | p.Gln61His | 40.20 |
| 22 | *KRAS* | 25398285 | c.34G>C | p.Gly12Arg | 46.00 |
| 23 | *KRAS* | 25398284 | c.35G>A | p.Gly12Asp | 43.19 |
| 24 | *KRAS* | 25398266 | c.53C>A | p.Ala18Asp | 47.59 |
| 25 | *KRAS* | 25398284 | c.35G>C | p.Gly12Ala | 34.63 |
| 26 | *KRAS*<br>*NF1* | 25380285<br>29496957 | c.112-17440C>T<br>c.528T>A | p.Thr58Ile<br>p.Asp176Glu | 55.06<br>37.34 |
| 27 | *CBL* | 119148991 | c.1211G>A | p.Cys404Tyr | 86.02 |
| 28 | *CBL* | 119148919 | c.1139T>C | p.Leu380Pro | 93.67 |
| 29 | *CBL* | 119148891 | c.1111T>C | p.Tyr371His | 89.49 |
| 30 | *CBL* | 119148925 | c.1145A>G | p.Lys382Arg | 74.40 |
| 31 | *CBL* | 119149246 | c.1254C>G | p.Phe418Leu | 92.30 |
| 32 | *CBL* | 119148991 | c.1211G>A | p.Cys404Tyr | 34.30 |
| 33 | *CBL* | 119148883 | c.1103A>G | p.Tyr368Cys | 32.21 |
| 34 | *NF1* | 29653035 | c.4970A>G | p.Tyr1657Cys | 87.90 |
| 35 | *NF1* | 29554236 | c.4956C>A | p.Asn1673Lys | 27.66 |
| 36 | *NF1* | 29496957 | c.630T>A | p.Asp176Glu | 49.44 |
| 37 | *NF1* | 29665757 | c.6855>A | p.Tyr2285* | 86.50 |
| 38 | *PTPN11* | 112940006 | c.1658C>T | p.Thr553Met | 52.86 |
| 39 | *PTPN11* | 112888165 | c.181G>T | p.Asp61Tyr | 34.83 |
| 40 | *PTPN11* | 112926910 | c.1530G>T | p.Gln510His | 50.43 |
| 41 | *PTPN11* | 112888202 | c.218C>T | p.Thr73Ile | 27.50 |
